# A modifier in the 129S2/SvPasCrl genome is responsible for the viability of *Notch1*[12f/12f] mice

**DOI:** 10.1186/s12861-019-0199-3

**Published:** 2019-10-07

**Authors:** Shweta Varshney, Hua-Xing Wei, Frank Batista, Mohd Nauman, Subha Sundaram, Katherine Siminovitch, Ankit Tanwar, Pamela Stanley

**Affiliations:** 1 Department of Cell Biology, Albert Einstein College of Medicine, New York, NY 10461 USA; 2 Present address: ETHOS Health Communications, Yardley, PA 19067 USA; 3 Present address: The First Affiliated Hospital of USTC, Division of Life Sciences and Medicine, University of Science and Technology of China, Hefei, Anhui 230001 People’s Republic of China; 4 Lunenfeld-Tanenbaum Research Institute, Mount Sinai Hospital, University of Toronto, Toronto, Ontario, CA M5G 1X5 Canada

**Keywords:** Notch signaling, Embryogenesis, *Notch1*[12f], Modifier gene, Backcross, C57BL/6J, 129S2/SvPasCrl

## Abstract

**Background:**

Mouse NOTCH1 carries a highly conserved O-fucose glycan at Thr466 in epidermal growth factor-like repeat 12 (EGF12) of the extracellular domain. O-Fucose at this site has been shown by X-ray crystallography to be recognized by both DLL4 and JAG1 Notch ligands. We previously showed that a *Notch1* Thr466Ala mutant exhibits very little ligand-induced NOTCH1 signaling in a reporter assay, whereas a Thr466Ser mutation enables the transfer of O-fucose and reverts the NOTCH1 signaling defect. We subsequently generated a mutant mouse with the Thr466Ala mutation termed *Notch1*[12f](*Notch1*^tm2Pst^). Surprisingly, homozygous *Notch1*[12f/12f] mutants on a mixed background were viable and fertile.

**Results:**

We now report that after backcrossing to C57BL/6 J mice for 11–15 generations, few homozygous *Notch1*[12f/12f] embryos were born. Timed mating showed that embryonic lethality occurred by embryonic day (E) ~E11.5, somewhat delayed compared to mice lacking *Notch1* or *Pofut1* (the O-fucosyltransferase that adds O-fucose to Notch receptors), which die at ~E9.5. The phenotype of C57BL/6 J *Notch1*[12f/12f] embryos was milder than mutants affected by loss of a canonical Notch pathway member, but disorganized vasculogenesis in the yolk sac, delayed somitogenesis and development were characteristic. In situ hybridization of Notch target genes *Uncx4.1* and *Dll3* or western blot analysis of NOTCH1 cleavage did not reveal significant differences at E9.5. However, qRT-PCR of head cDNA showed increased expression of *Dll3*, *Uncx4.1* and *Notch1* in E9.5 *Notch1*[12f/12f] embryos. Sequencing of cDNA from *Notch1*[12f/12f] embryo heads and Southern analysis showed that the *Notch1*[12f] locus was intact following backcrossing. We therefore looked for evidence of modifying gene(s) by crossing C57BL/6 J *Notch1* [12f/+] mice to 129S2/SvPasCrl mice. Intercrosses of the F1 progeny gave viable F2 *Notch1*[12f/12f] mice.

**Conclusion:**

We conclude that the 129S2/SvPasCrl genome contains a dominant modifying gene that rescues the functions of NOTCH1[12f] in signaling. Identification of the modifying gene has the potential to illuminate novel factor(s) that promote Notch signaling when an O-fucose glycan is absent from EGF12 of NOTCH1.

## Background

Canonical Notch signaling is induced by cell-cell interactions between a Notch receptor and a Notch ligand [1]. There are four mammalian Notch receptors (NOTCH1 to NOTCH4) and five Notch ligands (DLL1, DLL3, DLL4, JAG1 and JAG2). The extracellular domain (ECD) of Notch receptors comprises 29–36 EGF repeats that may be modified by O-glycans [2–4]. O-fucose glycans are initiated by the transfer of fucose from GDP-fucose by POFUT1 at Ser/Thr in the consensus sequence C_2_XXXXS/TC_3_ between the second and third cysteines of an EGF repeat [5]. There are 21 O-fucose glycan consensus sequences in the 36 EGF repeats of NOTCH1 and a similar number in the other Notch ECDs. Experiments in *Drosophila* determined that deletion of EGF11 and EGF12 destroys Notch ligand binding and defined a Notch ligand binding domain [6]. EGF12 of both *Drosophila* and mammalian Notch carry O-fucose [7, 8] and X-ray crystallography of co-crystals between fragments of NOTCH1 and DLL4 [9] as well as NOTCH1 and JAG1 [10] revealed that O-fucose on EGF12 plays a key role in binding interactions between NOTCH1 and both ligands.

Conversion of Thr to Ala in the EGF12 O-fucose consensus site of *Drosophila* Notch precludes the addition of O-fucose to EGF12, and results in embryos that go through neurogenesis but fail to exhibit Notch signaling at the dorsal/ventral boundary of the wing disc [11]. In vitro binding assays revealed that there is a marked loss in the ability of Fringe (which adds a N-acetylglucosamine to O-fucose in EGF repeats) to inhibit the binding of Serrate to Notch lacking O-fucose in EGF12 [11]. However, Fringe markedly enhances the binding of both Delta and Serrate to *Drosophila* Notch when O-fucose is absent from EGF12 [11]. Thus, when all other O-fucose sites remain intact and can be modified by Ofut1 and Fringe, the loss of O-fucose in EGF12 primarily has an effect on Serrate/Notch interactions. Interestingly, a recombinant mammalian NOTCH1 fragment containing EGF12 lacking O-fucose does bind to Notch ligands in vitro in the presence of calcium [12].

Notch signaling in mice is very similar to Notch signaling in humans. Since disrupted Notch signaling causes numerous human pathologies [13], defining mechanisms of Notch signaling in mice provides insights into human disease. Introduction of the Thr to Ala mutation (Thr466Ala) in EGF12 of a mouse *Notch1* cDNA causes the resulting mutant NOTCH1[12f] to be inactive in a Notch signaling reporter assay [14]. Reversion of the mutation in the mutant cDNA rescues Notch signaling. Introduction of a Ser in place of Ala in the *Notch1*[12f] cDNA allows the acquisition of O-fucose and the concomitant rescue of Notch signaling. Thus, in cultured mammalian cells, the presence of O-fucose on EGF12 is necessary for Notch signaling. Therefore, it was surprising to find that *Notch1*[12f/12f] mouse homozygotes are viable and fertile [15, 16]. The *Notch1*[12f] allele is hypomorphic, however, since it only partially rescues a compound heterozygous *Notch1*[12f/lbd] mouse [15]. *Notch1*[lbd] (*Notch1*^tm2.1Pst^) is a strong loss-of-function allele lacking 141 amino acids that include the NOTCH1 ligand binding domain [17, 18]. In this paper we show that *Notch1*[12f] becomes a strong loss-of-function allele after backcrossing at least 11 generations onto the C57BL/6 J background, and we reveal the existence of a modifier gene in either the 129129S2/SvPasCrl or C57BL/6J genome.

## Results

### C57BL/6 J *Notch1*[12f/12f] embryos die before birth

**Fig. 1:**
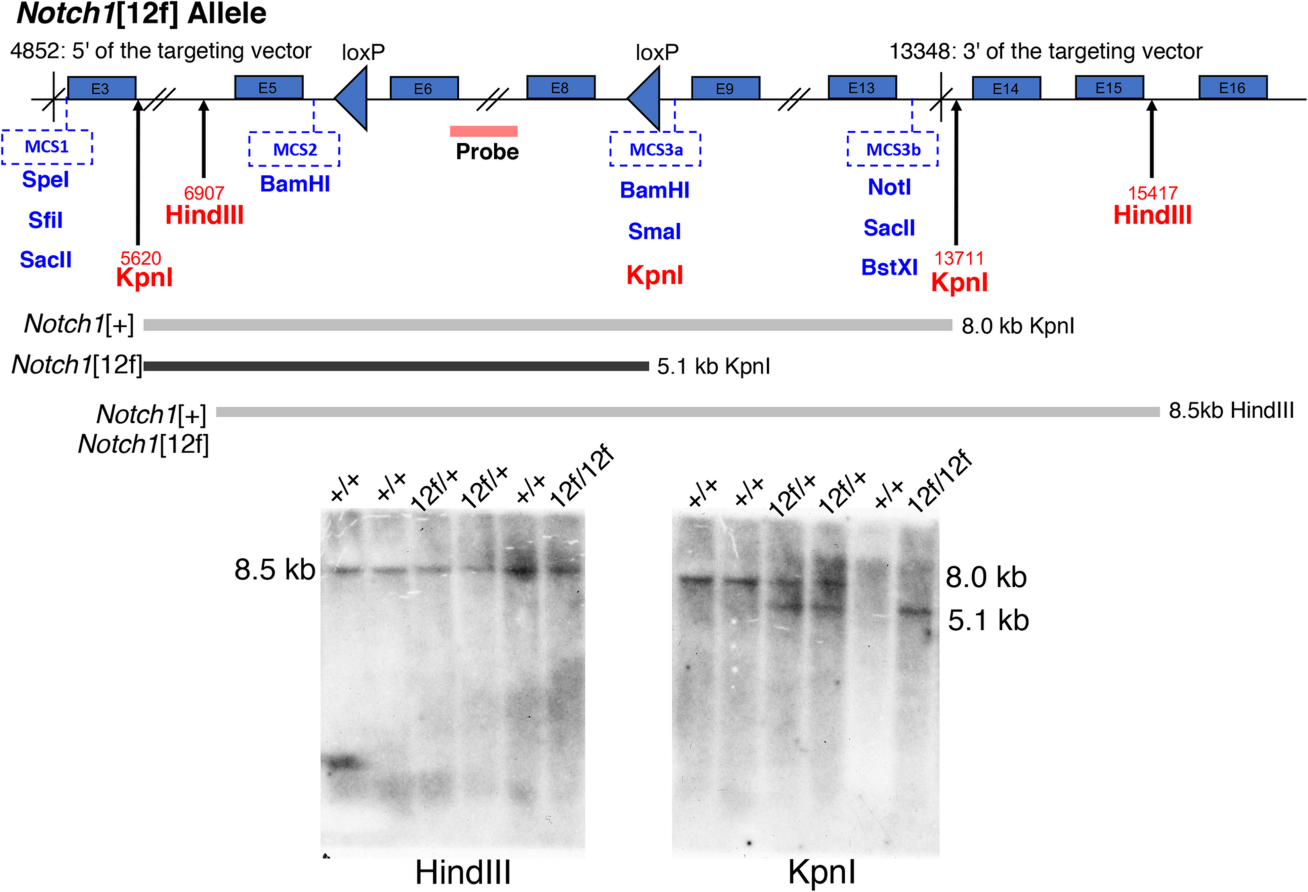
Southern blot analysis. Genomic DNA prepared from *Notch1*[+/+], *Notch1*[12f/+] and *Notch1*[12f/12f] progeny of a heterozygous Notch1[12f/+] cross, was digested with HindIII or KpnI, subjected to agarose gel electrophoresis, transferred to membrane and probed with the DNA fragment shown on the diagram of the *Notch1*[12f] allele. Numbered boxes represent introns. MCS, multiple cloning site of the targeting vector

The *Notch1*[12f] allele (*Notch1*^tm2Pst^) was originally characterized in mice of mixed genetic background. WW6 embryonic stem (ES) cells were engineered to carry a *Notch1* point mutation in exon 8 in a floxed targeting construct. WW6 ES cells are ~ 75% 129S2/SvPasCrl, ~ 20% C57BL/6 J and ~ 5% SJL [19]. Chimeric mice were crossed to C57BL/6 J MeuCre40 mice [20] to obtain the *Notch1*[12f] allele, and then crossed 5–7 generations to C57BL/6 J mice before heterozygotes were intercrossed and their progeny characterized [15]. This colony was maintained over several years by heterozygote crosses and progeny were obtained at the expected Mendelian frequency. Backcrossing of heterozygotes to C57BL/6 J males and females was subsequently performed in a separate cohort until *Notch1*[12f/+] heterozygotes of the eleventh backcross generation were intercrossed. When several pairs did not give rise to *Notch1*[12f/12f] homozygous progeny at the expected frequency (Table 1, 1^st^ cohort), we performed Southern analysis and quantitative PCR of genomic DNA of *Notch1*[12f/+] heterozygotes to determine if there were copy number or structural changes in the *Notch1*[12f] gene. Digestion with HindIII or Kpn1 gave bands of the expected size for *Notch1*[+] and *Notch1*[12f] alleles, with no additional bands (Fig. 1). Primers that spanned a *loxP* site in the *Notch1*[12f] allele and primers specific for the *Notch1*[+] allele were used to determine copy number in *Notch1*[+] and *Notch1*[12f] genomic DNA, as described in Methods. Heterozygous mice proven to carry one copy each of a wild type and a mutant allele, and Kpn1 bands from *Notch1*[+] and *Notch1*[12f] of equivalent intensity (Fig. 1) determined by NIH Image J, were used for timed mating of heterozygous Notch1[12f/+] mice.

**Table 1:** Embryonic lethality of *Notch1*[12f/12f] after backcrossing to C57BL/6 J

Cross	Intercross Pairs (Litters)	*Notch1* [+/+]	*Notch1* [12f/+]	*Notch1* [12f/12f]	Chi-Squared Test
Toronto *Notch1*[12f/+] X Toronto *Notch1*[12f/+]	5 (8)	18	36	14	
*Expected ratio*		17	34	17	*P* = 0.7
Backcrossed 11 generations (1st cohort)	19 (65)	103	178	13	
C57BL/6 J.*Notch1*[12f/+] X C57BL/6 J*.Notch1*[12f/+]					
*Expected ratio*		74	147	74	*P* = 1.57e-15
Backcrossed 15 generations (2nd cohort)	9 (9)	19	33	2	
C57BL/6 J.*Notch1*[12f/+] X C57BL/6 J*.Notch1*[12f/+]					
*Expected ratio*		14	27	14	*P = 0.0013*

We also reacquired *Notch1*[12f/12f] homozygotes from a colony established in Toronto from *Notch1*[12f/12f] males we sent. The latter were backcrossed 3 times to C57BL/6 J mice and maintained by intercrossing before sending us male homozygotes. These mice were used to independently replicate our published data [15] showing reduced numbers of thymocytes in *Notch1*[12f/12f] homozygotes (not shown). After crossing the *Notch1*[12f/12f] homozygotes from the Toronto colony once to C57BL/6 J, we used qPCR of genomic DNA to show that the resulting heterozygotes each had a single copy of *Notch1*[+] and *Notch1*[12f]. These mice were intercrossed and shown to generate viable *Notch1*[12f/12f] homozygotes at the expected Mendelian frequency (Table 1). Heterozygotes were separately backcrossed to C57BL/6 J for up to 15 generations and then intercrossed. As previously observed in our colony after 11 backcrosses, this second set of backcrossed *Notch1*[12f/+] heterozygotes rarely generated viable *Notch1*[12f/12f] homozygotes (Table 1, 2^nd^ cohort). Analysis of embryos from timed mating of *Notch1*[12f/+] heterozygotes revealed that embryonic lethality of C57BL/6 J *Notch1*[12f/12f] homozygotes occurred by E11.5 (Table 2).

**Table 2:** Stage of embryonic lethality in *Notch1*[12f/12f] embryos

Embryo Stage	No. Litters	Genotype		
		*Notch1* [+/+]	*Notch1* [12f/+]	*Notch1* [12f/12f]
E9.5	3	6	16	8 (1 dying)
E10.5	4	12	24	6 (1 dying)
E10.75	2	3	12	1
E11.5	3	5	15	4 (all dying)
E12.5	1	3	5	0

Timed mating was performed as described in Methods

### *Notch1*[12f/12f] embryos exhibit a milder phenotype than typical notch pathway mutants

**Fig. 2:**
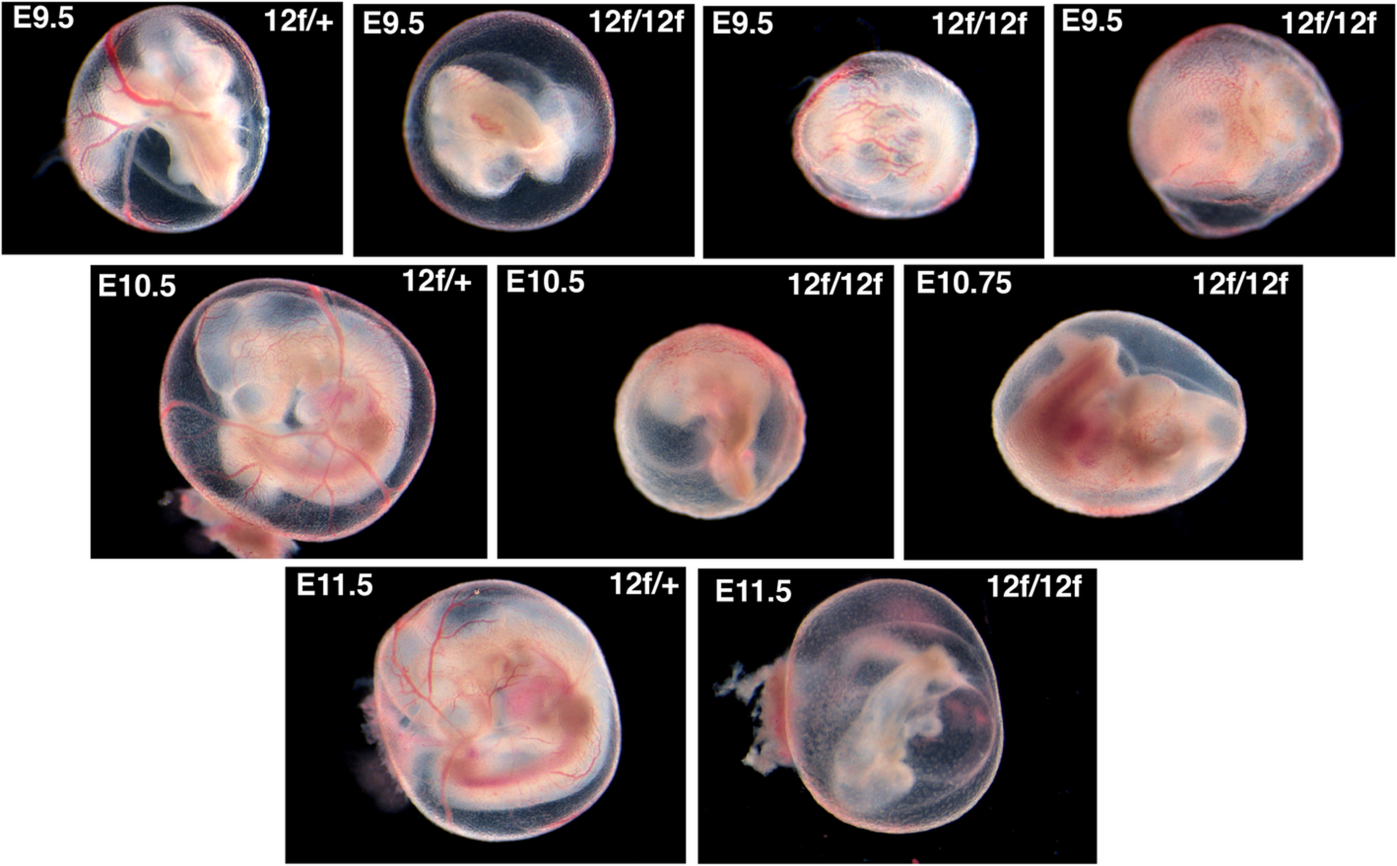
Yolk sac vascularization in *Notch1*[12f/12f] embryos. *Notch1*[12f/+] mice that had been backcrossed 11 generations to C57BL/6 J mice were subjected to timed mating as described in Methods, embryos were dissected at E9.5, E10.5 and E11.5 and photographed using an inverted dissecting microscope

**Fig. 3:**
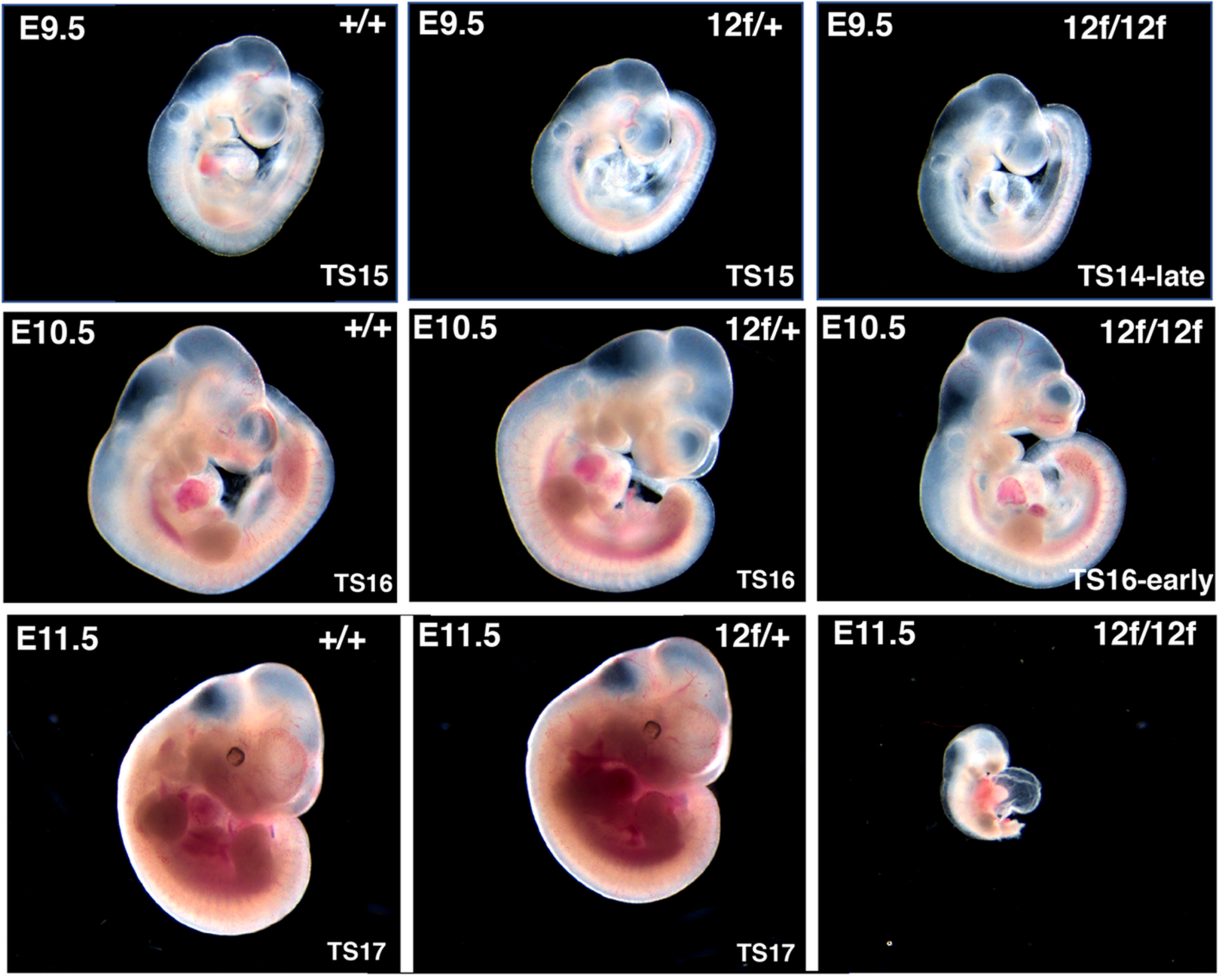
Morphology of *Notch1*[12f/12f] embryos. Embryos were obtained at E9.5, E10.5 and E11.5 as described in the legend to Fig. 2 and photographed under an inverted dissecting microscope. Their Theiler stage was determined by comparison of morphological features including number of somites, presence, delimitation and size of forelimb bud and hindlimb bud, branchial arch number and size, shape and size of the heart and head regions, olfactory placode indentation and closure of the otocyst. Other E9.5 and E10.5 embryos are shown in Additional files 1 and 2; Figures S1, S2

E9.5 mouse embryos with defective Notch signaling exhibit highly disorganized vascularization of the yolk sac [21–25]. In C57BL/6 J *Notch1*[12f/12f] embryos of E9.5 to E11.5, the yolk sac was poorly vascularized with major blood vessels ill-defined compared to control littermates which had a clear vascular tree with prominent blood vessels at each stage (Fig. 2). The overall morphology of wild type and heterozygous embryos was similar to *Notch1*[12f/12f] mutants at E9.5 (Fig. 3). However, based on comparisons of their Theiler stage (TS) [26], E9.5 *Notch1*[12f/12f] homozygotes were delayed in development compared to littermates. They had a significant reduction in the total number of somites at E9.5 (Table 3), mutant embryos were relatively anemic and close to TS14-late or TS15-early, whereas littermates were more similar to TS15-late (Fig. 3 and Additional file 1: Figure S1). By E10.5, mutant embryos were similarly less developed and more anemic than controls (Fig. 3 and Additional file 2: Figure S2), and by E11.5 dying mutants exhibited a strong Notch signaling-defective phenotype with an enlarged pericardial sac and small heart, severely truncated somites and general regression of most tissues (Fig. 3). In general, overall body size of *Notch1*[12f/12f] homozygotes was smaller, features were less defined, the heart and mandibular arches were generally smaller and limb buds less developed. Staining with anti-PECAM1 of E10.5 embryos highlights these features (Additional file 3: Figure S3). The staining intensity was somewhat reduced in *Notch1*[12f/12f] embryos and vascularization appeared slightly more sparse.

**Table 3:** Number of somites in *Notch1*[12f/12f] embryos

E9.5 Embryos	No. Somites	Mean (SEM)	Student’s t test^a^
*Notch1*[+/+] (*n* = 5)	23, 24, 25, 24, 23	24 (+/− 0.37)	
*Notch1*[12f/12f] (*n* = 4)	21, 20, 20, 19	20 (+/− 0.41)	*P* = 0.0003

^a^Unpaired Student’s t test with Welch’s correction

To determine whether a mutation of the *Notch1*[12f] gene might have occurred during backcrossing that could explain the embryonic lethality of *Notch1*[12f/12f] embryos, cDNA from wild type and E9.5 homozygous mutant embryo heads was prepared. Sequencing of cDNAs obtained from PCR products using 12 overlapping primers that spanned the entire coding region (Additional file 4: Table S1) gave the expected mutant sequence of *Notch1*[12f] cDNA, and no indication of a mixed sequence. The *Notch1* sequence obtained from *Notch1*[12f/12f] embryos contained the nucleotide changes introduced to generate the *Notch1*[12f] (T466A) knock-in allele and the introduced *Sph*1 restriction site [15]. Thus, no mutation or rearrangement had inactivated the *Notch1* gene during backcrossing of *Notch1*[12f/+] mice to the 15th generation.

**Fig. 4:**
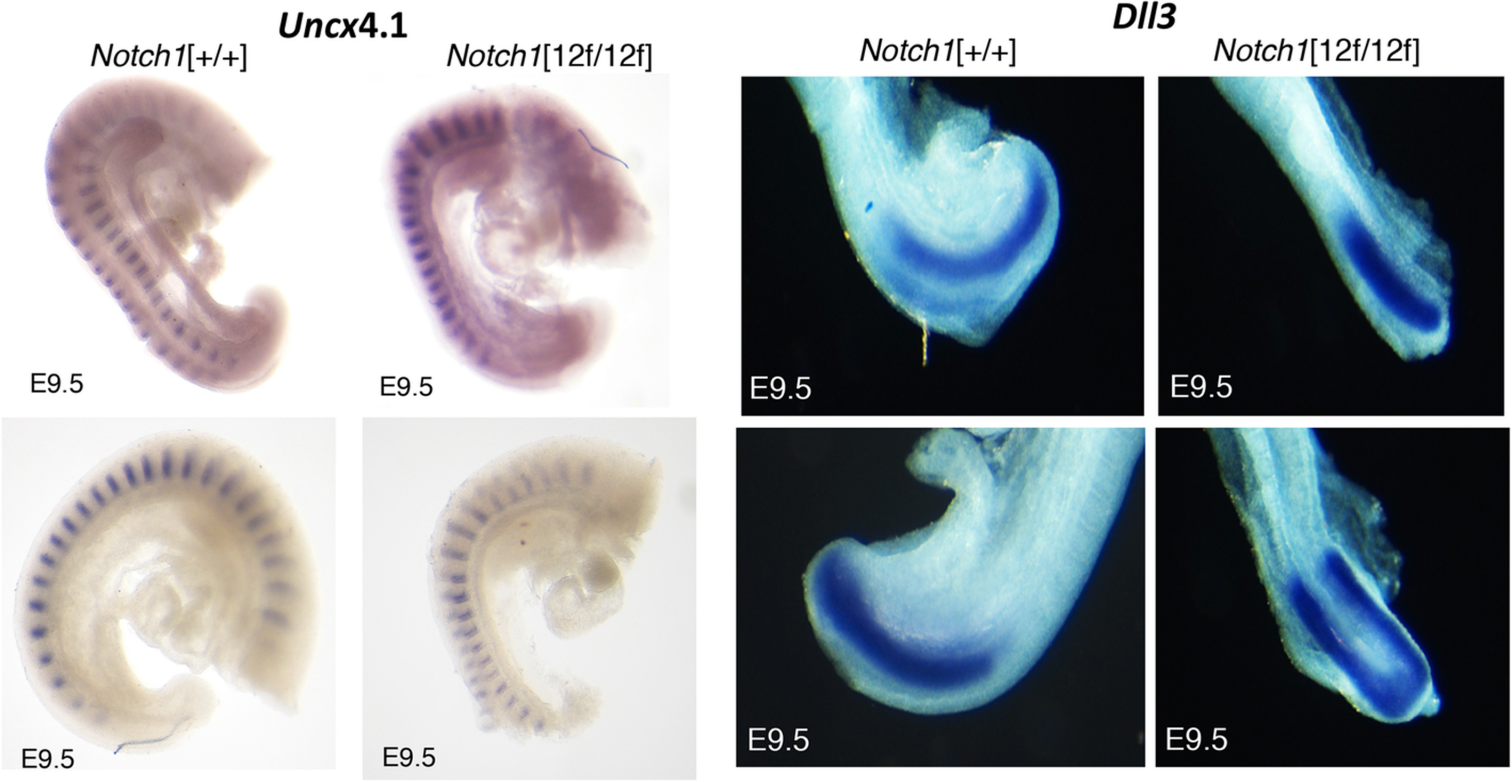
In situ hybridization of E9.5 embryos. The somitic region of embryos obtained at E9.5 was subjected to in situ hybridization using a probe for *Uncx*4.1 or *Dll3*

**Fig. 5:**
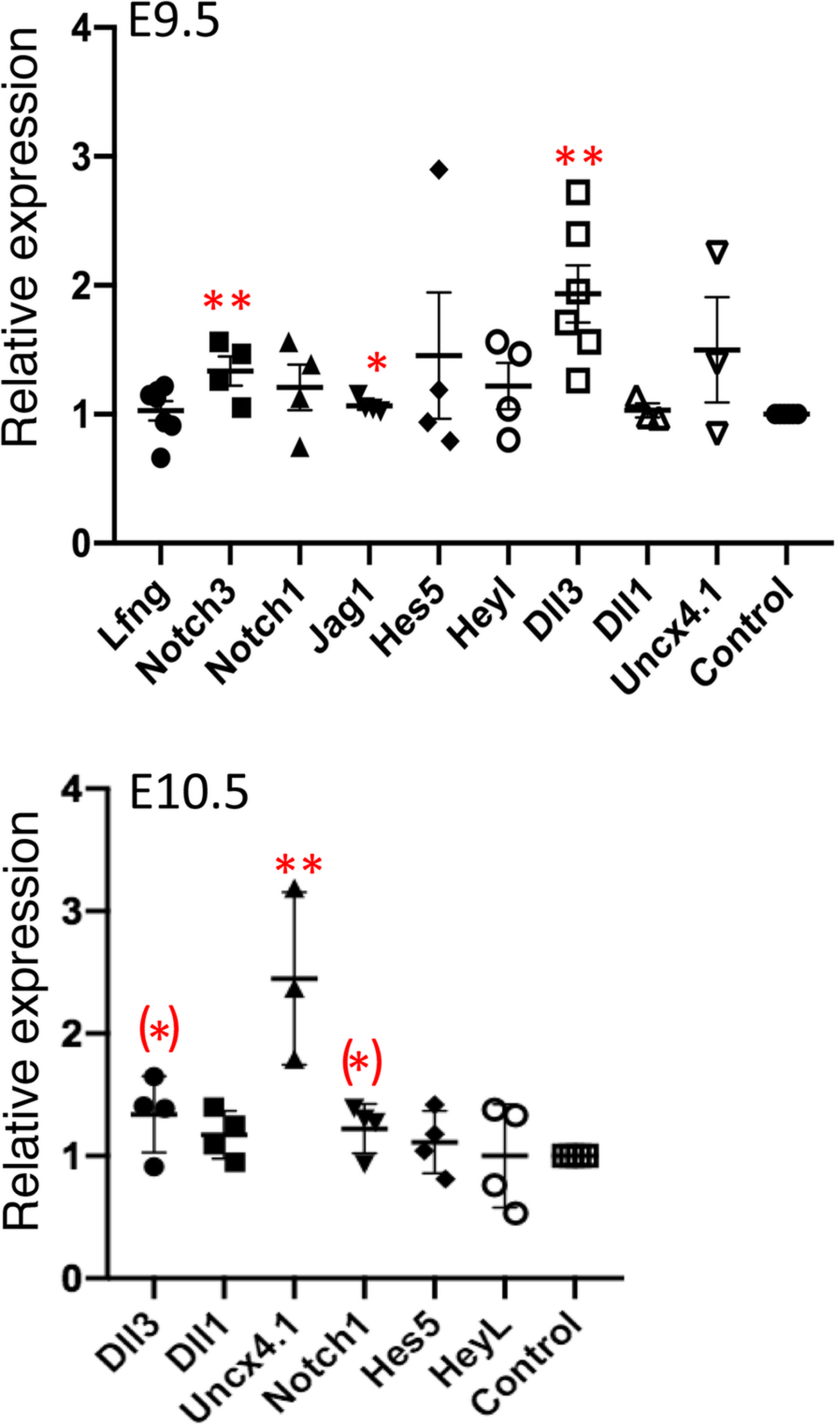
qRT-PCR of Notch pathway genes expressed by *Notch1*[12f/12f] embryos. RNA from E9.5 and E10.5 embryo and control heads (*n* = 3 of each) was converted to cDNA and tested in replicate qRT-PCR experiments using *Actb* and *Gapdh* housekeeping genes. Values for mutants and controls compared to each housekeeping gene were obtained and fold-difference between mutants and controls (taken as 1.0) was determined. The data were plotted using Prism version 8.0. Significance was determined by unpaired Student’s t test, (*) *p* = 0.07; * *p* < 0.05; ** *p* < 0.005

To determine if Notch signaling was reduced in the presomitic mesoderm (PSM), in situ hybridization of *Uncx*4.1 and *Dll3* was performed. Previous studies of Notch pathway mutants have shown reduced expression of these genes at E9.5 [21, 27, 28]. However, we observed similar signals for control and mutant embryos at E9.5 (Fig. 4). Western blot analysis to detect cleaved NOTCH1 intracellular domain (NICD1) as an indicator of Notch1 signaling was performed on the PSM of E10.5 embryos. The results showed variable NICD1 expression which did not indicate reduced cleavage in *Notch1*[12f/12f] embryos (Additional file 5: Figure S4). A subset of Notch pathway genes whose expression was previously shown by in situ hybridization to increase in defined brain regions of Notch-signaling defective *Pofut1* null embryos [21], was investigated by qRT-PCR of the cDNA from *Notch1*[12f/12f] embryo heads. At E9.5, *Notch1*, *Jag1* and *Dll3* expression were significantly increased in *Notch1*[12f/12f] compared to wild type embryo transcripts in replicate experiments performed in triplicate using two housekeeping genes (Fig. 5), and in a Notch pathway qRT-PCR array using five housekeeping genes (not shown). In E10.5 embryos, *Uncx4.1* was significantly increased based on qRT-PCR analysis with *Actb* and *Gapdh* housekeeping genes (Fig. 5).

### Reversal of *Notch1*[12f/12f] lethality in a mixed genetic background

To investigate the existence of a modifying gene that might have been lost during backcrossing, 15 generation backcrossed *Notch1*[12f/+] male and female mice were crossed to wild type 129S2/SvPasCrl mice. This strain was chosen because WW6 ES cells from which the original *Notch1[*12f/12f] mice were derived are ~ 75% 129S2/SvPasCrl [19]. The resulting *Notch1*[12f/+] F1 progeny of mixed C57BL/6 J and 129S2/SvPasCrl background were intercrossed and the F2 progeny were genotyped. It is shown in Table 4 that *Notch1*[12f/12f] pups from the F2 intercross appeared at the expected Mendelian ratio of ~ 25%. Thus, a single outcross rescued the embryonic lethality of *Notch1*[12f/12f] embryos, providing strong evidence for the existence of a dominant modifying gene (or tightly-linked genes) outside the congenic footprint that rescues NOTCH1[12f] function in the C57BL/6 J:129S2/SvPasCrl mixed genetic background. The congenic footprint is defined as the genes that remain linked to *Notch1*[12f] after the 11~15 backcrosses to C57BL/6 J mice [29]. It is expected that these genes would derive from the predominant 129S2/SvPasCrl genome of WW6 cells [29].

**Table 4:** Intercross of *Notch1*[12f/+] F1 progeny obtained from the cross C57BL/6 J.*Notch1*[12f/+] X 129S2/SvPasCrl.*Notch1*[+/+]

Cross	No. Pairs (No. Litters)	*Notch1* [+/+]	*Notch1* [12f/+]	*Notch1* [12f/12f]	Chi-Squared test
C57BL/6 J.129S2/SvPasCrl *Notch1*[12f/+] F1 X C57BL/6 J.129S2/SvPasCrl *Notch1*[12f/+] F1	6 (11)	27	42	24	
*Expected*		23	47	23	*P* = 0.58

## Discussion

In this paper we show that *Notch1*[12f/12f] homozygotes that are viable on a mixed background of C57BL/6 J and 129S2/SvPasCrl, die at ~E11.5 after 11–15 generations of backcrossing to C57BL/6 J mice. This phenomenon occurred after backcrossing heterozygotes derived from two colonies that were producing viable *Notch1*[12f/12f] homozygotes. The phenotype of C57BL/6 J *Notch1*[12f/12f] embryos resembles that of embryos with defective Notch signaling [21–25]. The yolk sac of *Notch1*[12f/12f] embryos exhibits fewer and disorganized blood vessels. Embryos of E9.5 have a slight reduction in the number of somites and are at an earlier Theiler stage of development than littermate controls. Homozygous embryos are no longer found by E12.5 and dying embryos at E11.5 are severely underdeveloped. Thus, *Notch1*[12f/12f] embryos live a day, or more, longer than embryos that lack NOTCH1 signaling [21–25]. The basis of their less severe phenotype in the developing embryo may be the presence of the many remaining O-glycans on Notch EGF repeats when only the EGF12 O-fucose glycan is absent [3].

The chance of loci outside the congenic footprint of the *Notch1*[12f] gene being heterozygous after 15 generations of backcrossing is (1/2)^15–1^ or 0.000061 [30]. It is expected that the congenic footprint derives from the 129S2/SvPasCrl genome since it is predominant in WW6 ES cells. The fact that a single intercross between C57BL/6 J/129S2/SvPasCrl F1 heterozygotes produced viable *Notch1*[12f/12f] pups in the expected Mendelian ratio suggests that a 129S2/SvPasCrl gene (or tightly linked genes) outside the congenic footprint is responsible for the viability of F2 *Notch1*[12f/12f] homozygotes. The fact that the loss of this modifying gene during backcrossing was not evident after ~ 7 backcrosses, when less than 1% of the genome would be 129S2/SvPasCrl, is surprising. However, both the viability of *Notch1*[12f/12f] mice from intercrosses after ~ 7 backcrosses, and the loss of viability after backrossing reached 11 or more generations, were observed in two cohorts maintained separately. This apparent paradox indicates that the “viability” gene may be linked to the *Notch1*[12f] allele on chromosome 2, though outside the congenic footprint.

The potential function(s) encoded by the 129S2/SvPasCrl modifying gene are myriad because the Notch signaling pathway is so complex [21–25]. It requires correct synthesis of a large membrane receptor, modification, processing and trafficking through the secretory pathway to the cell surface, stable expression at the cell surface, interaction with canonical ligands and endocytosis into the ligand-expressing cell. While there are only 5 canonical NOTCH1 ligands, there are numerous membrane and secreted proteins that bind to NOTCH1 ECD and may affect canonical Notch signaling [31]. A modifier might be important for NOTCH1[12f] dimerization or oligomerization. We previously reported genetic evidence that *Notch1*[12f] compound heterozygotes with a null NOTCH1 ligand binding domain allele (*Notch1*[lbd] or *Notch1*^tm2.1Pst^) have a Notch developmental phenotype, whereas *Notch1*[lbd/+] heterozygotes do not, indicating cell surface interactions between the products of the *Notch1*[12f] and *Notch1*[lbd] alleles [18]. Evidence from electron microscopy suggests that *Drosophila* Notch and mammalian NOTCH1, respectively, dimerize through their extracellular domains [32]. A modifier could encode a molecule that assists transit of NOTCH1[12f] from the endoplasmic reticulum through Golgi compartments, or provides stability at the plasma membrane, or modifies NOTCH1[12f] through a post-translational mechanism to interact with Notch ligands. Previously identified modifiers of Notch signaling include Fringe which modulates the strength of Notch signaling via canonical Notch ligands [33], DNER, a non-canonical Notch ligand [34] and molecules of a variety of activities identified by genetic interactions [35–37] or modifier screens [38]. An example of mouse background influencing a Notch signaling phenotype is *Jag1* mutant heterozygotes that exhibit a more severe inner ear phenotype on a C3H background compared to a C57BL/6 background, and eye dysmorphologies that manifest on a C57BL/6 background but are suppressed on a C3H background [39]. Identifying the mechanism by which NOTCH1[12f] becomes functional will require isolating the modifying gene and showing how the activity it encodes enables NOTCH1[12f] to participate in Notch signaling.

The most straight-forward way to identify a modifier would be a SNP genotyping strategy on a large cohort of 129S2/SvPasCrl/C57BL/6 J *Notch1*[12f/12f] F2 progeny to locate a region of 129S2/SvPasCrl homology present in all progeny. This strategy was successful in genomic mapping of several recessive mutant genes with phenotypic traits using a small panel of 394 SNPs and a relatively modest number of F2 progeny [40]. However, the regions mapped in that study varied from 17 to 83 Mb. By contrast, the Affymetrix Mouse Diversity Genotyping Array uses 63,000 SNPs with a potential of mapping to a region as small as 4.6 kb. A complementary strategy would be to transfect a range of cell types with the mouse *Notch1*[12f] cDNA shown previously to be inactive in ligand-induced Notch signaling in CHO cells [14], identify a host cell that allows NOTCH1[12f] to function, and isolate the modifying gene from a cDNA expression library made from that cell. This would be facilitated if the gain-of-function phenotype was easy to screen for or for which to select. While each of these approaches would require considerable effort, it would potentially unearth a novel mechanism by which NOTCH1 with an inactivating point mutation that precludes the addition of O-fucose in the canonical Notch ligand binding domain can become functional. Such a mechanism may be in operation in cell types that do not completely lose Notch signaling when *Pofut1* is deleted. Examples include the inner ear [41], hemopoietic stem cells [42], and endocardial cells [43].

## Conclusion

We conclude that the viability of *Notch1*[12f/12f] homozygotes on the mixed genetic background generated by 5–7 backcrosses to C57BL/6 J mice after derivation of the targeted allele in WW6 ES cells, is due to the presence of a gene in the 129S2/SvPasCrl background. This modifier gene allows the production of viable *Notch1*[12f/12f] mice by rescuing NOTCH1[12f] function in Notch signaling.

## Methods

### Mice

*Notch1*[12f](Notch1^tm2Pst^) mice were generated previously in WW6 embryonic stem (ES) cells by a targeted knockin strategy [15]. C57BL/6 J mice were obtained from The Jackson Laboratory (Bar Harbor, MN) and 129S2/SvPasCrl mice were obtained from Charles River Laboratories, USA. Mice of both sexes and various ages from 7 weeks to 9 months were used in breeding. Mice were housed at a maximum of 5 mice per cage containing autoclaved 1/8th inch Bed-o’Cobs™ (Andersons Lab Bedding, Delphi, Indiana) in the mouse barrier facilities at the Albert Einstein College of Medicine (temperature 68° to 72 °F, humidity 30–70%, lights on at 6 am and off at 8 pm daily). Mice were fed ad libitum with LabDiet 5058 (PicoLab mouse diet 20).

Timed mating was performed to obtain embryos at different stages of gestation. A time of 0.5 days of gestation was taken as the first morning that a vaginal plug was observed in a female who had been placed with a male the evening before. Mice were sacrificed using asphyxiation to effect in a chamber containing CO_2_ delivered from a compressed gas cylinder, followed by cervical dislocation. Mice were bred in the animal facilities at the Albert Einstein College of Medicine and at the University Health Network (UHN) in Toronto, and sacrificed using CO_2_ followed by cervical dislocation under protocols respectfully approved by the Institutional Animal Care and Use Committee (IACUC) of the Albert Einstein College of Medicine (protocols 20080813, 20110803, 20140803 and 20170709) and the UHN Animal Care Committee.

After backcrossing 5–7 times to C57BL/6 J, the colony was maintained for several years by intercrossing heterozygotes and *Notch1*[12f/12f] homozygote progeny were viable and fertile. Following backcrossing to 11 or more generations, however, *Notch1*[12f/12f] homozygotes generally died during gestation. We analyzed a large number of embryos from this 1st cohort. We then reacquired viable *Notch1*[12f/12f] mice that we had previously sent to Toronto and which had been backcrossed to C57BL/6 for 3 generations and then intercrossed to derive [12f/12f] homozygotes. These mice were crossed once to C57BL/6 J mice at Albert Einstein and then heterozygotes were intercrossed to maintain a colony that generated viable *Notch1*[12f/12f] mice. Other *Notch1*[12f/12f] mice from Toronto were backcrossed to C57BL/6 J mice to 15 generations before heterozygotes were intercrossed. This colony was maintained as *Notch1*[12f/+] heterozygotes because *Notch1*[12f/12f] homozygotes were not viable. We analyzed numerous embryos from this 2nd cohort. 129S2/SvPasCrl mice were used to cross to the latter *Notch1*[12f/+] heterozygotes.

Genotyping of progeny was performed by PCR of genomic DNA from toe or yolk sac using forward primer Fwd-CGGAGTGACGGGTATGTAT and reverse primer Rev-GTGTGTGTTGTGGCAGGTTC which span a *lox p* site between exons 5 and 6 of the mouse *Notch1* locus [15]. The *Notch1*[+] allele product was 458 bp and the *Notch1*[12f] allele product was 561 bp. Timed mating was performed to determine stage of embryonic death and to generate embryos for analysis. A time of 0.5 days of gestation was taken as the first morning that a vaginal plug was observed in a female who had been placed with a male the evening before. Embryos were staged according to established Theiler staging criteria [26].

### Southern blot analysis

Genomic DNA was prepared, digested with HindIII or KpnI, electrophoresed on an agarose gel, transferred to membrane and hybridized to a ^32^P-labeled probe as previously described [15, 44]. The probe was generated from genomic DNA with forward primer 5’AGTCACCTGTGGTTCCCTTG and reverse primer 5’AAGGGATGGGGAGAGAGAAA, gel purified and labeled with α-^32^P-dCTP using the Prime-It RmT Random Primer hybridization labeling kit (Agilent Technologies). After development, films were scanned and quantitated by NIH ImageJ.

### In situ hybridization

In situ hybridization probes were prepared and used to perform in situ hybridization as described previously [21, 45]. Anti-PECAM1 antibody (BD Pharminogen, MEC13.3) binding to whole mount embryos was also performed as described previously [18].

### qPCR and qRT-PCR

To determine copy number of *Notch1*[+] and *Notch1*[12f] alleles a qPCR strategy was employed. A standard number-of-molecules curve was performed with a plasmid containing a 100 bp fragment of the serum albumin precursor gene *Alb* and used in subsequent experiments to calculate the number of molecules in each sample. Numbers between 1,000 and 1,000,000 were within the linear range. Genomic DNA from wild type, *Notch1*[12f/+] and *Notch1*[12f/12f] progeny of *Notch1*[12f/+] heterozygote crosses was subjected to qPCR using the following primers: *Alb* Fwd-CAGGTGTCAACCCCAACTCT; Rev-CCACACAAGGCAGTCTCTGA; *Notch1*[+] Fwd-GGTTCTTGTGAGCTATTAGCAGCT; Rev-AAGATGAGCTCCCTGCTCTG; *Notch1*[12F] Fwd-TCCAGTTGGAGTCAGCACAG; Rev-CAGCCCAACCTGATCCTCTA. The amount of product from each allele compared to *Alb* was calculated and the ratio of alleles was determined and compared to a wild type C57BL/6 J genomic DNA standard.

To determine expression of Notch target genes, wild type and *Notch1*[12f/12f] embryos at E9.5 were genotyped from yolk sac genomic DNA and their heads were dissected for extraction of RNA using the Trizol method (Invitrogen). RNA amount was determined by nanodrop and 250 ng was reverse transcribed using a Verso cDNA kit (Thermo Fischer Scientific cDNA kit). Primers for *Notch1* RT-PCR (Additional file 4: Table S1) and for qRT-PCR of Notch target genes (Additional file 6: Table S2) were designed using NCBI Primer blast. PCR products of overlapping *Notch1* primers were generated and sent for sequencing by Macrogen. Primers used in qRT-PCR were shown to give single amplification products in an Applied Biosystems Viia 7 thermal cycler. Real time PCR was performed using 1 μl of the cDNA and Absolute Blue qPCR mix (Thermo Fischer Scientific). PCR conditions were 50 °C for 2 min, 95 °C for 10 min, followed by 40 cycles of 95 °C for 15 s, 60 °C for 1 min, 95 °C for 15 s and 60 °C for 1 min. Gene expression relative to housekeeping genes (*Actb*, *Gapdh*) was calculated using the 2^-ddCt^ method as described [46]. A Notch1 pathway RT^2^ Profiler PCR PAMM-259Z array (Qiagen) was also used to determine relative gene expression in control versus *Notch1*[12f/12f] cDNA preparations from embryonic brain. The array included 5 housekeeping genes.

### Statistics

The significance of mouse progeny expected versus observed was determined by Chi-squared test. Numeric data were analyzed for significance using the unpaired Student’s t test assuming both populations have the same STDEV for qRT-PCR or using Welch’s correction for comparison of the number of somites in Prism, version 8.0, as noted.

## Supplementary information

**Additional file 1:** **Figure S1.** Morphology of E9.5 *Notch1*[12f/12f] embryos. Embryos were obtained at E9.5 and photographed under an inverted dissecting microscope. Their Theiler stage was determined by comparison of morphological features including number of somites, presence, delimitation and size of forelimb bud and hindlimb bud, branchial arch number and size, shape and size of the heart and head regions, olfactory placode indentation and closure of the otocyst.

**Additional file 2:** **Figure S2.** Morphology of E10.5 *Notch1*[12f/12f] embryos. Embryos were obtained at E10.5 and photographed under an inverted dissecting microscope. Their Theiler stage was determined by comparison of morphological features including number of somites, presence, delimitation and size of forelimb bud and hindlimb bud, branchial arch number and size, shape and size of the heart and head regions, olfactory placode indentation and closure of the otocyst.

**Additional file 3:** **Figure S3.** Vascularization of Notch1[12f/12f] embryos. Embryos were isolated at E10.5, their yolk sacs removed for genotyping, the tip of the PSM was removed for western analysis (see Additional file 4; Figure S4). Staining of fixed embryos with anti-PECAM1 antibody was performed as described previously [1].1. Ge C, Stanley P: Effects of varying Notch1 signal strength on embryogenesis and vasculogenesis in compound mutant heterozygotes. *BMC Dev Biol* 2010, 10(1):36.

**Additional file 4:** **Table S1.** Primers used to sequence *Notch*12f mRNA, NM_008714.3

**Additional file 5:** **Figure S4**. Notch1 activation in the presomitic mesoderm (PSM) of E10.5 embryos. The tip of the PSM was removed from a cohort of E10.5 embryos (see Additional file 3; Figure S3) and transferred to 40 μl Laemmli buffer, sonicated in a sonicating water bath for 5 min, heated at 90 °C for 15 min and frozen at − 20 °C. After thawing, 20 μl was analyzed by SDS-PAGE. After electrophoresis the gel was transferred to PVDF membrane, the membrane was cut at ~ 75 kDa and the upper portion was probed with anti-Val1744 Ab (Cell Signaling) for cleaved, activated NOTCH1 (NICD1) and binding was detected with HRP-conjugated anti-rabbit IgG. A positive control sample with NICD1 was included on the gel. GAPDH on the bottom portion was detected with primary mAb 5718 (R & D Systems) and HRP-conjugated anti-mouse IgG. The membrane was treated with Pierce West-Pico 34,095 ECL reagent for 5 min and exposed to film for various times. A 10 min exposure is shown. A replicate western blot with the same samples gave the same results.

**Additional file 6:** **Table S2.** Primers used in qRT-PCR

## Data Availability

All data generated or analyzed during this study are included in this published article or are available on request.

## References

[1] Kovall RA, Gebelein B, Sprinzak D, Kopan R (2017). The canonical notch signaling pathway: structural and biochemical insights into shape, sugar, and force. Dev Cell.

[2] Takeuchi H, Haltiwanger RS (2014). Significance of glycosylation in notch signaling. Biochem Biophys Res Commun.

[3] Varshney S, Stanley P (2018). Multiple roles for O-glycans in notch signalling. FEBS Lett.

[4] Harvey BM, Haltiwanger RS (2018). Regulation of notch function by O-glycosylation. Adv Exp Med Biol.

[5] Wang Y, Shao L, Shi S, Harris RJ, Spellman MW, Stanley P, Haltiwanger RS (2001). Modification of epidermal growth factor-like repeats with O-fucose. Molecular cloning and expression of a novel GDP-fucose protein O- fucosyltransferase. J Biol Chem.

[6] Rebay I, Fleming RJ, Fehon RG, Cherbas L, Cherbas P, Artavanis-Tsakonas S (1991). Specific EGF repeats of notch mediate interactions with Delta and serrate: implications for notch as a multifunctional receptor. Cell.

[7] Harvey BM, Rana NA, Moss H, Leonardi J, Jafar-Nejad H, Haltiwanger RS (2016). Mapping sites of O-glycosylation and fringe elongation on Drosophila notch. J Biol Chem.

[8] Kakuda S, Haltiwanger RS (2017). Deciphering the fringe-mediated notch code: identification of activating and inhibiting sites allowing discrimination between ligands. Dev Cell.

[9] Luca VC, Jude KM, Pierce NW, Nachury MV, Fischer S, Garcia KC (2015). Structural basis for Notch1 engagement of Delta-like 4. Science.

[10] Luca VC, Kim BC, Ge C, Kakuda S, Wu D, Roein-Peikar M, Haltiwanger RS, Zhu C, Ha T, Garcia KC (2017). Notch-jagged complex structure implicates a catch bond in tuning ligand sensitivity. Science.

[11] Lei L, Xu A, Panin VM, Irvine KD (2003). An O-fucose site in the ligand binding domain inhibits notch activation. Development.

[12] Cordle J, Redfieldz C, Stacey M, van der Merwe PA, Willis AC, Champion BR, Hambleton S, Handford PA (2008). Localization of the delta-like-1-binding site in human Notch-1 and its modulation by calcium affinity. J Biol Chem.

[13] Aster JC (2014). In brief: notch signalling in health and disease. J Pathol.

[14] Shi S, Ge C, Luo Y, Hou X, Haltiwanger RS, Stanley P (2007). The threonine that carries fucose, but not fucose, is required for Cripto to facilitate nodal signaling. J Biol Chem.

[15] Ge C, Stanley P (2008). The O-fucose glycan in the ligand-binding domain of Notch1 regulates embryogenesis and T cell development. Proc Natl Acad Sci U S A.

[16] Visan I, Yuan JS, Liu Y, Stanley P, Guidos CJ (2010). Lunatic fringe enhances competition for delta-like notch ligands but does not overcome defective pre-TCR signaling during thymocyte beta-selection in vivo. J Immunol.

[17] Ge C, Liu T, Hou X, Stanley P (2008). In vivo consequences of deleting EGF repeats 8-12 including the ligand binding domain of mouse Notch1. BMC Dev Biol.

[18] Ge C, Stanley P (2010). Effects of varying Notch1 signal strength on embryogenesis and vasculogenesis in compound mutant heterozygotes. BMC Dev Biol.

[19] Ioffe E, Liu Y, Bhaumik M, Poirier F, Factor SM, Stanley P (1995). WW6: an embryonic stem cell line with an inert genetic marker that can be traced in chimeras. Proc Natl Acad Sci U S A.

[20] Leneuve P, Colnot S, Hamard G, Francis F, Niwa-Kawakita M, Giovannini M, Holzenberger M (2003). Cre-mediated germline mosaicism: a new transgenic mouse for the selective removal of residual markers from tri-lox conditional alleles. Nucleic Acids Res.

[21] Shi S, Stanley P (2003). Protein O-fucosyltransferase 1 is an essential component of notch signaling pathways. Proc Natl Acad Sci U S A.

[22] Krebs LT, Xue Y, Norton CR, Shutter JR, Maguire M, Sundberg JP, Gallahan D, Closson V, Kitajewski J, Callahan R (2000). Notch signaling is essential for vascular morphogenesis in mice. Genes Dev.

[23] Conlon RA, Reaume AG, Rossant J (1995). Notch1 is required for the coordinate segmentation of somites. Development.

[24] Swiatek PJ, Lindsell CE, del Amo FF, Weinmaster G, Gridley T (1994). Notch1 is essential for postimplantation development in mice. Genes Dev.

[25] Copeland JN, Feng Y, Neradugomma NK, Fields PE, Vivian JL (2011). Notch signaling regulates remodeling and vessel diameter in the extraembryonic yolk sac. BMC Dev Biol.

[26] Armit C, Richardson L, Hill B, Yang Y, Baldock RA (2015). eMouseAtlas informatics: embryo atlas and gene expression database. Mamm Genome.

[27] Feller J, Schneider A, Schuster-Gossler K, Gossler A (2008). Noncyclic notch activity in the presomitic mesoderm demonstrates uncoupling of somite compartmentalization and boundary formation. Genes Dev.

[28] Takahashi Y, Inoue T, Gossler A, Saga Y (2003). Feedback loops comprising Dll1, Dll3 and Mesp2, and differential involvement of Psen1 are essential for rostrocaudal patterning of somites. Development.

[29] Schalkwyk LC, Fernandes C, Nash MW, Kurrikoff K, Vasar E, Koks S (2007). Interpretation of knockout experiments: the congenic footprint. Genes Brain Behav.

[30] Silver LM (1995). Mouse genetics. Concepts and applications.

[31] Wang MM (2011). Notch signaling and notch signaling modifiers. Int J Biochem Cell Biol.

[32] Kelly DF, Lake RJ, Middelkoop TC, Fan HY, Artavanis-Tsakonas S, Walz T (2010). Molecular structure and dimeric organization of the notch extracellular domain as revealed by electron microscopy. PLoS One.

[33] Irvine KD (1999). Fringe, notch, and making developmental boundaries. Curr Opin Genet Dev.

[34] Eiraku M, Tohgo A, Ono K, Kaneko M, Fujishima K, Hirano T, Kengaku M (2005). DNER acts as a neuron-specific notch ligand during Bergmann glial development. Nat Neurosci.

[35] Muller R, Jenny A, Stanley P (2013). The EGF repeat-specific O-GlcNAc-transferase Eogt interacts with notch signaling and pyrimidine metabolism pathways in Drosophila. PLoS One.

[36] Calpena Eduardo, López del Amo Víctor, Chakraborty Mouli, Llamusí Beatriz, Artero Rubén, Espinós Carmen, Galindo Máximo I. (2017). TheDrosophila junctophilingene is functionally equivalent to its four mammalian counterparts and is a modifier of a Huntingtin poly-Q expansion and the Notch pathway. Disease Models & Mechanisms.

[37] Lau LF (2016). Cell surface receptors for CCN proteins. J Cell Commun Signal.

[38] Shalaby NA, Parks AL, Morreale EJ, Osswalt MC, Pfau KM, Pierce EL, Muskavitch MA (2009). A screen for modifiers of notch signaling uncovers Amun, a protein with a critical role in sensory organ development. Genetics.

[39] Kiernan AE, Li R, Hawes NL, Churchill GA, Gridley T (2007). Genetic background modifies inner ear and eye phenotypes of jag1 heterozygous mice. Genetics.

[40] Moran JL, Bolton AD, Tran PV, Brown A, Dwyer ND, Manning DK, Bjork BC, Li C, Montgomery K, Siepka SM (2006). Utilization of a whole genome SNP panel for efficient genetic mapping in the mouse. Genome Res.

[41] Basch ML, Brown RM, Jen HI, Semerci F, Depreux F, Edlund RK, Zhang H, Norton CR, Gridley T, Cole SE, et al. Fine-tuning of notch signaling sets the boundary of the organ of Corti and establishes sensory cell fates. ELife. 2016;5:1-23. 10.7554/eLife.19921 PMC5215100 27966429

[42] Wang W, Yu S, Zimmerman G, Wang Y, Myers J, Yu VW, Huang D, Huang X, Shim J, Huang Y (2015). Notch receptor-ligand engagement maintains hematopoietic stem cell quiescence and niche retention. Stem Cells.

[43] Wang Y, Wu B, Lu P, Zhang D, Wu B, Varshney S, Del Monte-Nieto G, Zhuang Z, Charafeddine R, Kramer AH (2017). Uncontrolled angiogenic precursor expansion causes coronary artery anomalies in mice lacking Pofut1. Nat Commun.

[44] Southern EM (1975). Detection of specific sequences among DNA fragments separated by gel electrophoresis. J Mol Biol.

[45] Chen J, Lu L, Shi S, Stanley P (2006). Expression of notch signaling pathway genes in mouse embryos lacking b4galactosyltransferase-1. Gene Expr Patterns.

[46] Livak KJ, Schmittgen TD (2001). Analysis of relative gene expression data using real-time quantitative PCR and the 2(−Delta Delta C(T)) method. Methods.

