# Supplementary figures and images for: A modifier in the 129S2/SvPasCrl genome is responsible for the viability of Notch1[12f/12f] mice
Source: BMC Dev Biol. 2019 Oct 7;19:19. doi: 10.1186/s12861-019-0199-3 (PMC6781419; doi:10.1186/s12861-019-0199-3)

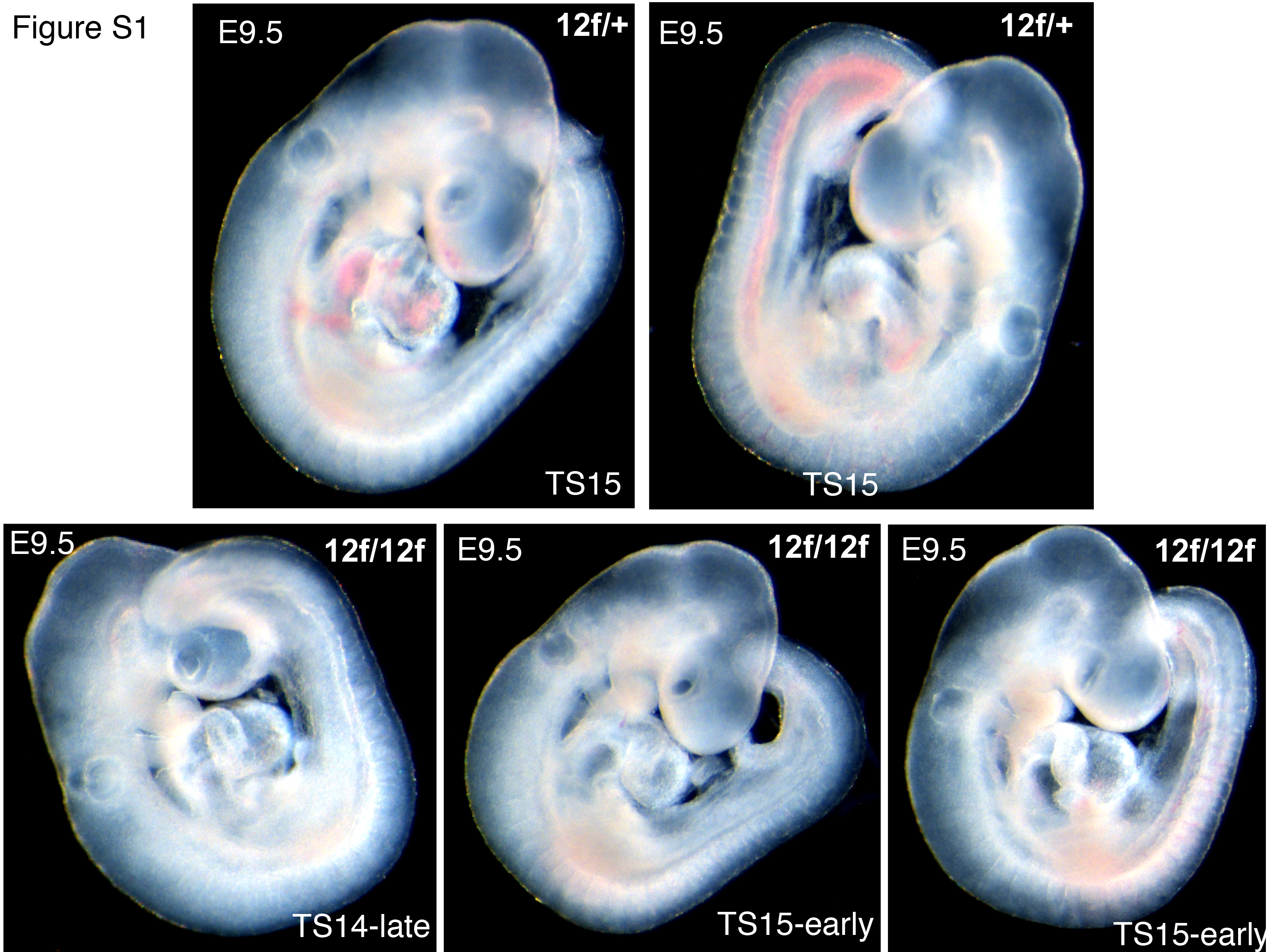

Supplement: Supplementary file 1 — Additional file 1: Figure S1. Morphology of E9.5 Notch1[12f/12f] embryos. Embryos were obtained at E9.5 and photographed under an inverted dissecting microscope. Their Theiler stage was determined by comparison of morphological features including number of somites, presence, delimitation and size of forelimb bud and hindlimb bud, branchial arch number and size, shape and size of the heart and head regions, olfactory placode indentation and closure of the otocyst. [file 12861_2019_199_MOESM1_ESM.tif]

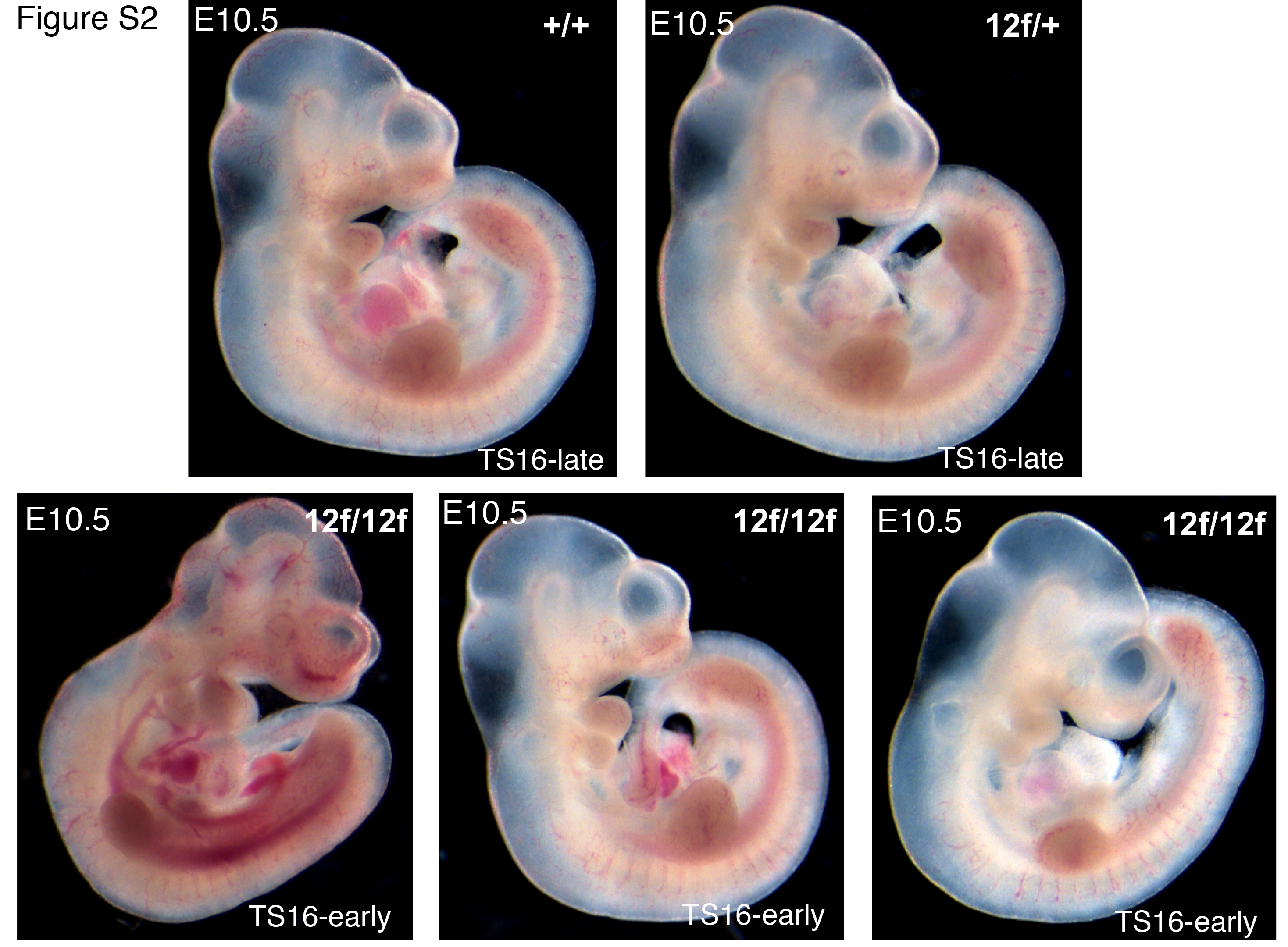

Supplement: Supplementary file 2 — Additional file 2: Figure S2. Morphology of E10.5 Notch1[12f/12f] embryos. Embryos were obtained at E10.5 and photographed under an inverted dissecting microscope. Their Theiler stage was determined by comparison of morphological features including number of somites, presence, delimitation and size of forelimb bud and hindlimb bud, branchial arch number and size, shape and size of the heart and head regions, olfactory placode indentation and closure of the otocyst. [file 12861_2019_199_MOESM2_ESM.tif]

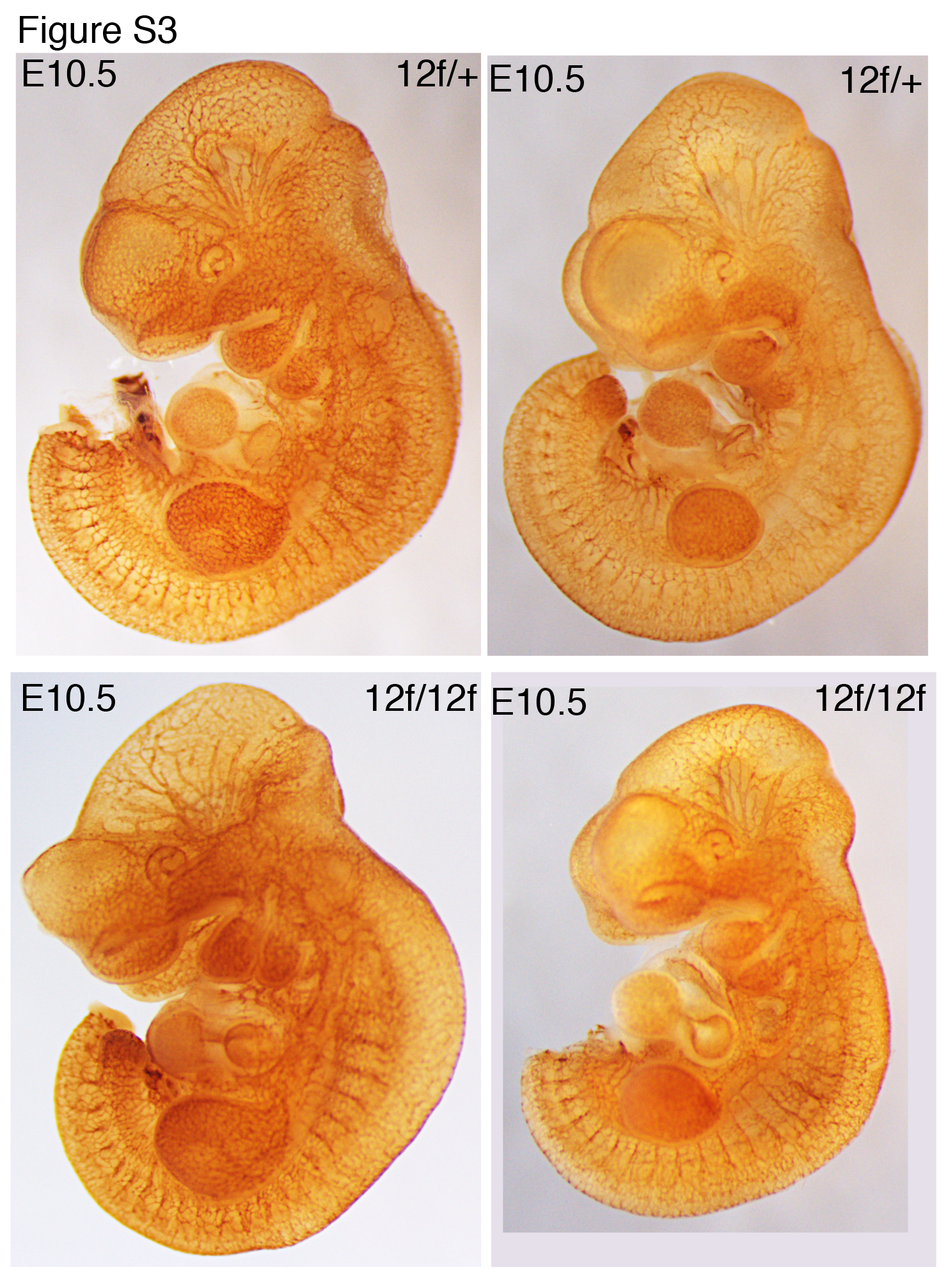

Supplement: Supplementary file 3 — Additional file 3: Figure S3. Vascularization of Notch1[12f/12f] embryos. Embryos were isolated at E10.5, their yolk sacs removed for genotyping, the tip of the PSM was removed for western analysis (see Additional file 4; Figure S4). Staining of fixed embryos with anti-PECAM1 antibody was performed as described previously [1].1. Ge C, Stanley P: Effects of varying Notch1 signal strength on embryogenesis and vasculogenesis in compound mutant heterozygotes. BMC Dev Biol 2010, 10(1):36. [file 12861_2019_199_MOESM3_ESM.tif]

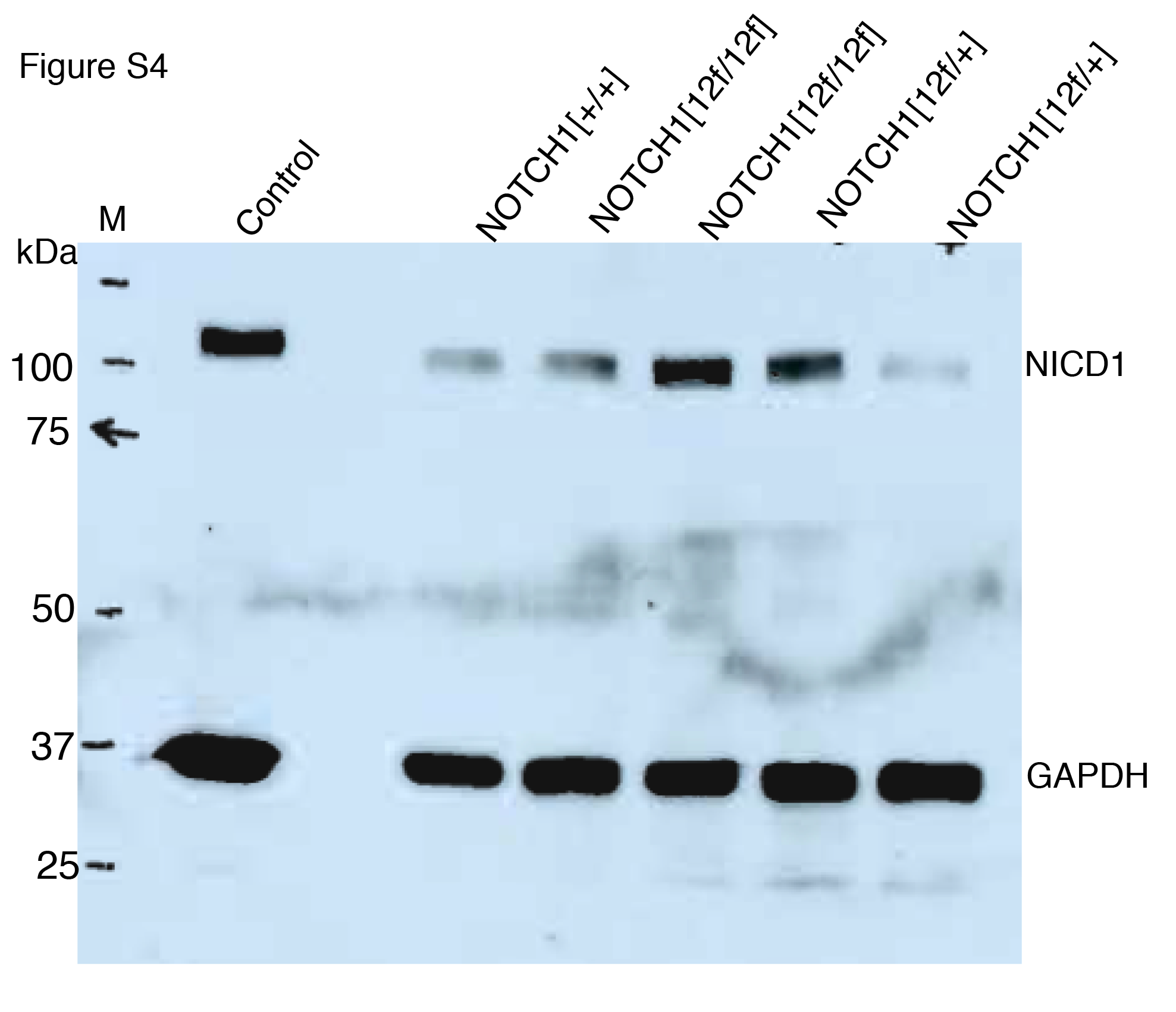

Supplement: Supplementary file 5 — Additional file 5: Figure S4. Notch1 activation in the presomitic mesoderm (PSM) of E10.5 embryos. The tip of the PSM was removed from a cohort of E10.5 embryos (see Additional file 3; Figure S3) and transferred to 40 μl Laemmli buffer, sonicated in a sonicating water bath for 5 min, heated at 90 °C for 15 min and frozen at − 20 °C. After thawing, 20 μl was analyzed by SDS-PAGE. After electrophoresis the gel was transferred to PVDF membrane, the membrane was cut at ~ 75 kDa and the upper portion was probed with anti-Val1744 Ab (Cell Signaling) for cleaved, activated NOTCH1 (NICD1) and binding was detected with HRP-conjugated anti-rabbit IgG. A positive control sample with NICD1 was included on the gel. GAPDH on the bottom portion was detected with primary mAb 5718 (R & D Systems) and HRP-conjugated anti-mouse IgG. The membrane was treated with Pierce West-Pico 34,095 ECL reagent for 5 min and exposed to film for various times. A 10 min exposure is shown. A replicate western blot with the same samples gave the same results. [file 12861_2019_199_MOESM5_ESM.tif]
