# Supplementary material for: A modifier in the 129S2/SvPasCrl genome is responsible for the viability of Notch1[12f/12f] mice
Source: BMC Dev Biol. 2019 Oct 7;19:19. doi: 10.1186/s12861-019-0199-3 (PMC6781419; doi:10.1186/s12861-019-0199-3)
Supplement: Supplementary file 4 — Additional file 4: Table S1. Primers used to sequence Notch12f mRNA, NM_008714.3 [file 12861_2019_199_MOESM4_ESM.docx]

|  | Position in *Notch1* | Product | Sequence | |
| --- | --- | --- | --- | --- |
|  | | | | |
| 1st set | Starts at 190 | 1085 bp | F | 5’-TGTGCGTCAACGTCCGATCC-3’ |
|  | ends at 1274 |  | R | 5’-TCAATGTTCTCACTGCAGTCCTCG-3’ |
|  | | | | |
| 2nd set | Starts at 1046 | 1050 bp | F | 5’-GTCCAGGAAACAACTGCAAGAATGGG-3’ |
|  | ends at 2095 |  | R | 5’-GTTGGCTGTGGCACTCATTGATGTT-3’ |
|  | | | | |
| 3rd set | Starts at 1865 | 1000 bp | F | 5’-GCACACCATGCAAGAACGGTG-3’ |
|  | ends at 2864 |  | R | 5’-TCGCAGGTTTGACCTTGCCA-3’ |
|  | | | | |
| 4th set | Starts at 2687 | 1078 bp | F | 5’-ATGATGTCGCTGGATACAAGTGCAAC-3’ |
|  | ends at 3764 |  | R | 5’-AAGCCGCCGAGATAGTCAGTG-3’ |
|  | | | | |
| 5th set | Starts at 3565 | 1000 bp | F | 5’-TGTGAGGTGGCTGCACAGAAG-3’ |
|  | ends at 4564 |  | R | 5’-CTGTGAAGCTGTAGTCCAGGATGTG-3’ |
|  | | | | |
| 6th set | Starts at 4254 | 1011 bp | F | 5’-TGGATTCATCTGTAGGTGCCCTGC-3’ |
|  | ends at 5264 |  | R | 5’-TAGACAATGGAGCCACGGATGTCC-3’ |
|  | | | | |
| 7th Set | Starts at 5032 | 1033 bp | F | 5’-TTCCACTTTCTGCGGGAGCTCA-3’ |
|  | ends at 6040 |  | R | 5’-CAGCATCTGAACGAGAGTATCGGGC-3’ |
|  | | | | |
| 8th Set | Starts at 5831 | 1034 bp | F | 5’-AGGTGGATGCTGACTGCATGG A-3’ |
|  | ends at 6864 |  | R | 5’-CAAGTAGCCATGGGGTGACTCG-3’ |
|  | | | | |
| 9th Set | Starts at 6634 | 1031 bp | F | 5’-ACTCTGTCTCCCACACTCTGCTC-3’ |
|  | ends at 7664 |  | R | 5’-GAGGAGTAACTGTGCTGGGAAGGAG-3’ |
|  | | | | |
| 10th set | Starts at 7390 | 1001 bp | F | 5’-ACTTACAGCTCCAGCCTCAGAACC-3’ |
|  | ends at 8373 |  | R | 5’-CATGAACCCAAGGTTCAAGGTCTGG-3’ |
|  | | | | |
| 11th set | Starts at 8139 | 1025 bp | F | 5’-TTTCCTGGTCAGGGTGAAGTTCCC-3’ |
|  | ends at 9163 |  | R | 5’-GCCATCCTGGGTTGTGCTCTTA-3’ |
|  | | | | |
| 12th set | Starts at 8825 | 665 bp | F | 5’-AAACAGAAGCTATGTGGCCTCTGAT-3’ |
|  | ends at 9489 |  | R | 5’-GTTTAGCAAAATGTGGACAACCATCAGTATC-3’ |

**Table S1.** Primers used to sequence *Notch*12f mRNA, NM_008714.3
