# Supplementary material for: A modifier in the 129S2/SvPasCrl genome is responsible for the viability of Notch1[12f/12f] mice
Source: BMC Dev Biol. 2019 Oct 7;19:19. doi: 10.1186/s12861-019-0199-3 (PMC6781419; doi:10.1186/s12861-019-0199-3)
Supplement: Supplementary file 6 — Additional file 6: Table S2. Primers used in qRT-PCR [file 12861_2019_199_MOESM6_ESM.docx]

| *Actb* | Forward | 5'- TTCTACAATGAGCTGCGTGTG -3' |
| --- | --- | --- |
|  | Reverse | 5'- GGGGTGTTGAAGGTCTCAAA -3’ |
| *Gapdh* | Forward | 5’-AAGGTCATCCCAGAGCTGAA-3’ |
|  | Reverse | 5’-CTGCTTCACCACCTTCTTGA-3’ |
| *Lfng* | Forward | 5’-CTGCACCATTGGCTACATTG-3’ |
|  | Reverse | 5’-ATGGGTCAGCTTCCACAGAG-3’ |
| *Notch3* | Forward | 5’-TGCAGTCAGCTGAGAATGACCACT-3’ |
|  | Reverse | 5’-ACATCCCGAAGTGGGTATGGGAAA-3’ |
| *Notch1* | Forward | 5’-CCCTTGCTCTGCCTAACGC-3’ |
|  | Reverse | 5’-GGAGTCCTGGCATCGTTGG-3’ |
| *Jag1* | Forward | 5’-TAGTGAATGTGCCCTGGTGTCCAT-3’ |
|  | Reverse | 5’ATGATCCTAAGGCTGCCATCACCA-3’ |
| *Hes5* | Forward | 5’-TACCTGAAACACAGCAAAGC-3’ |
|  | Reverse | 5’- GCTGGAAGTGGTAAAGCAG-3’ |
| *HeyL* | Forward | 5’-AGCATAGTCCCAATCCCACCATGT-3’ |
|  | Reverse | 5’-TGGTTGTGGGAAGTCAGCTCAGAA-3’ |
| *Dll3* | Forward | 5’-TCTTGGTCATCCACGTTCGC-3’ |
|  | Reverse | 5’-CCAGCACCGTCTTGTAACCT-3’ |
| *Dll1* | Forward | 5'-GGCTCTTCCCCTTGTTCTAAC-3' |
|  | Reverse | 5'-CTTCAGCCGGACGCAGACC-3' |
| *Uncx4.1* | Forward | 5’-ACCCGCACCAACTTTACCG-3’ |
|  | Reverse | 5’-TGGAACCAGACCTGAACTCG-3’ |

**Table S2.** Primers used in qRT-PCR
